# Defined three-dimensional culture conditions mediate efficient induction of definitive endoderm lineage from human umbilical cord Wharton’s jelly mesenchymal stem cells

**DOI:** 10.1186/s13287-016-0426-9

**Published:** 2016-11-16

**Authors:** Ashraf Al Madhoun, Hamad Ali, Sarah AlKandari, Valerie Lopez Atizado, Nadeem Akhter, Fahd Al-Mulla, Maher Atari

**Affiliations:** 1 Research Division, Dasman Diabetes Institute, 1180 Dasman, Kuwait; 2 Department of Medical Laboratory Sciences, Faculty of Allied Health Sciences, Health Sciences Center, Kuwait University, Al-Jabriya, Kuwait; 3 Department of Pathology, Molecular Pathology Unit, Faculty of Medicine, Health Sciences Center, Kuwait University, Al-Jabriya, Kuwait; 4 UIC Regenerative Medicine Research Institute, International University of Catalonia, Barcelona, Spain

**Keywords:** Wharton’s jelly, Wharton’s jelly-derived mesenchymal stem cells, Definitive endoderm, 3D, CXCR4, Sox17, Mesenchymal stromal cells

## Abstract

**Background:**

Wharton’s jelly-derived mesenchymal stem cells (WJ-MSCs) are gaining increasing interest as an alternative source of stem cells for regenerative medicine applications. Definitive endoderm (DE) specification is a prerequisite for the development of vital organs such as liver and pancreas. Hence, efficient induction of the DE lineage from stem cells is crucial for subsequent generation of clinically relevant cell types. Here we present a defined 3D differentiation protocol of WJ-MSCs into DE cells.

**Methods:**

WJ-MSCs were cultured in suspension to generate spheroids, about 1500 cells each, for 7 days. The serum-free differentiation media contained specific growth factors, cytokines, and small molecules that specifically regulate signaling pathways including sonic hedgehog, bone morphogenetic protein, Activin/Wnt, and Notch.

**Results:**

We obtained more than 85 % DE cells as shown with FACS analysis using antibodies directed against the DE marker CXCR4. In addition, biochemical and molecular analysis of bona-fide DE markers revealed a time-course induction of Sox17, CXCR4, and FoxA2. Focused PCR-based array also indicated a specific induction into the DE lineage.

**Conclusions:**

In this study, we report an efficient serum-free protocol to differentiate WJ-MSCs into DE cells utilizing 3D spheroid formation. Our approach might aid in the development of new protocols to obtain DE-derivative lineages including liver-like and pancreatic insulin-producing cells.

**Electronic supplementary material:**

The online version of this article (doi:10.1186/s13287-016-0426-9) contains supplementary material, which is available to authorized users.

## Background

Wharton’s jelly-derived mesenchymal stem cells (WJ-MSCs) have attracted tremendous interest in recent years as a potential stem cell source for both research and therapeutic applications because they displayed a high capacity for self-renewal, multilineage differentiation, and immune-modular properties in earlier studies [1–3]. This unique type of cells residing in the gelatinous insulator of blood vessels in the umbilical cord, named Wharton’s Jelly [4], have been studied extensively in the past decade and have been differentiated in vitro into a wide spectrum of cell types representing the three germ layers [1, 5–7].

In particular, endodermic differentiation gained growing attention in the field because its derived tissues such as pancreatic and hepatic tissues are heavily affected by diseases and pathological conditions. The potential of hepatic-like and pancreatic-like cell types from stem cells holds significant potential not only for regenerative medicine applications but also for drug testing and toxicology studies. Pluripotent stem cells (PSCs) such as embryonic stem cells (ESCs) and induced pluripotent stem cells (iPSCs) have been successfully differentiated into definitive endoderm (DE) and their derived lineages following extensive in-vitro induction protocols. These protocols have been designed to guide the cells through the developmental events by mimicking the embryonic conditions from the primitive streak stage to the DE stage and its derivative’s lineages [8–11].

The endodermal potential of WJ-MSCs has not been well established to date because only limited information has been reported on their endodermal differentiation capacity. Bhandari et al. [7] reported successful differentiation of WJ-MSCs into DE, pancreatic foregut, pancreatic endoderm, and β-cells following a 1-week differentiation protocol. Another group managed to differentiate WJ-MSCs into insulin-producing cells in vitro [12]. Other groups reported the differentiation of WJ-MSCs into insulin-producing cells with islet-like morphology, using recombinant adenovirus–*PDX1* gene constructs [13, 14]. Despite showing positive indications toward DE differentiation, these studies reported the use of animal serum and/or genetic modifications, and resulted in low differentiation capacities. Using stem cells, adherence to clinical scale standards requires genomic modification of the free cell type, and the development of highly efficient differentiation protocols free from animal products and chemically defined with detailed acknowledgment of the small molecules used to mediate differentiation.

The ability to direct WJ-MSCs efficiently to the DE lineage is a c**r**ucial step toward the development of downstream endodermic cells, such as hepatic or pancreatic β-like cells. WJ-MSCs can overcome the limitations of PSCs such as tumorigenicity, especially when considering potential clinical applications [15]. In addition, WJ-MSCs possess hypoimmunogenicity that makes this cell type a good candidate for potential allogenic therapeutic usages [3, 16, 17].

In this study, we present a novel three-dimensional (3D), fully defined, serum-free, stepwise differentiation protocol to generate DE from WJ-MSCs. Our 7-day culture condition utilizes the manipulation of several signaling pathways. Initially, the activation and inhibition of RA/KGF and SHH/BMP signaling, respectively, generated mesendoderm (ME) cells. The second step utilizes T3, EGF signaling induction, and the inhibition of TGF-β/Notch pathways to induce the DE lineage. This approach resulted in the enrichment of cells expressing DE markers by day 7. Further, our results demonstrate that WJ-MSCs can provide an excellent platform for DE generation.

## Methods

### Ethical approval and procurement of human samples

The study was approved by the Ethical Review Committee at the Dasman Diabetes Institute (protocol number: RA-2013-009) in accordance with the World Medical Association Declaration of Helsinki Ethical Principles for Medical Research Involving Human Subjects and Samples. Human umbilical cord matrix Wharton’s jelly mesenchymal stem cells (WJ-MSCs) were purchased from ATCC (PCS-500-010). We have previously characterized WJ-MSCs and showed that the cells are self-renewable, express stemness protein markers, and have multilineage differentiation properties including adipogenesis, chondrogenesis, and osteogenesis [1].

### WJ-MSC culture and maintenance

WJ-MSCs were maintained in DMEM/Hams’s F-12 (1:1 vol/vol) culture medium supplemented with 10 % MSC-qualified FBS, penicillin (100 units/ml), and streptomycin (100 μg/ml). Cell culture media and supplements were purchased from Invitrogen. Cell proliferation was monitored; upon reaching 70 % confluence, cells were detached using 0.05 % trypsin/0.02 % EDTA in PBS for the experimental procedure [1].

### 3D spheroidal colony formation and differentiation assay

**Fig. 1:**
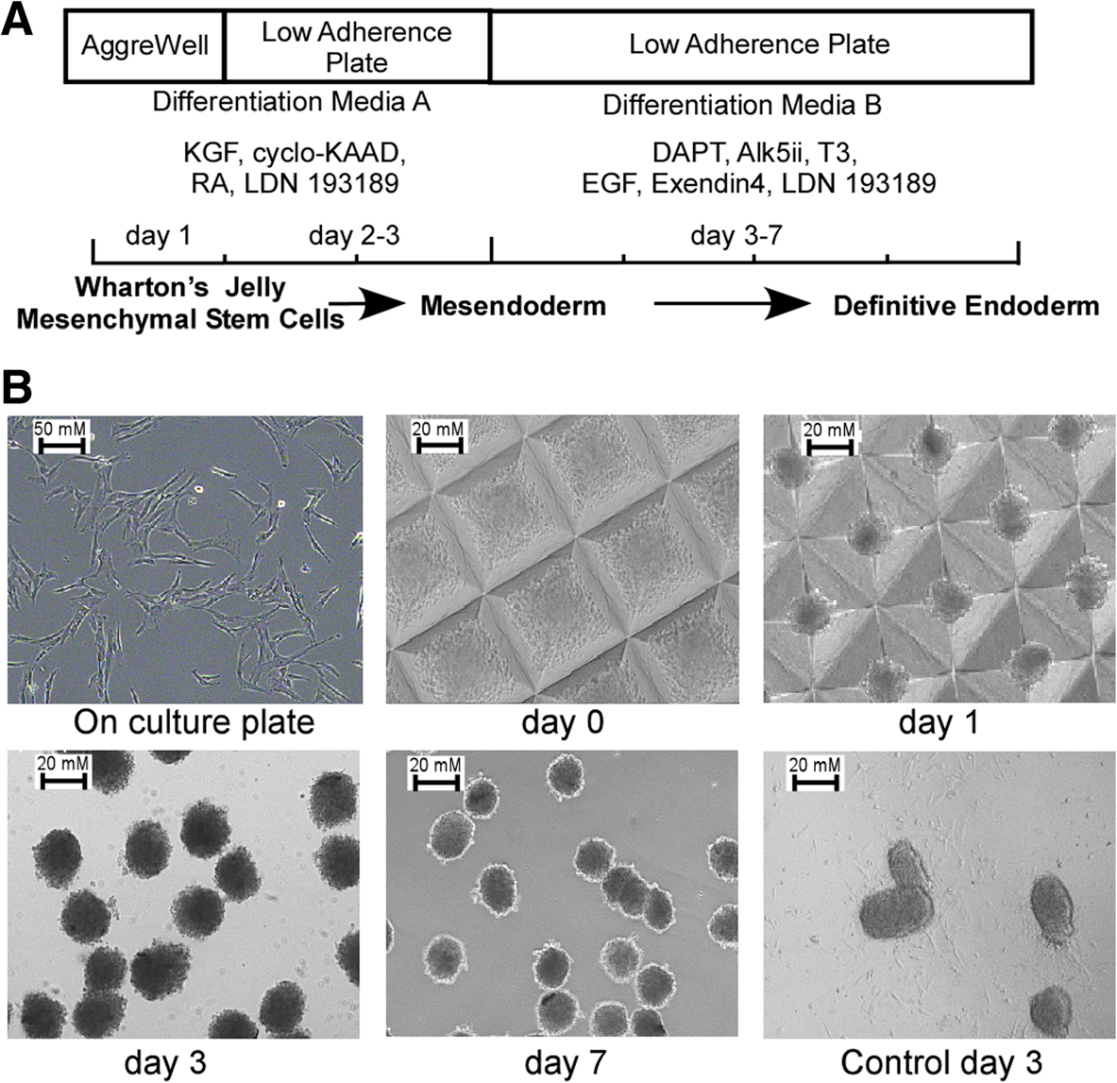
Experimental protocol and 3D colony formation. **a** Schematic representation of the differentiation protocol including the key manipulated signaling pathways. **b** Phase-contrast representative microscope images (Magnification x 200) for WJ-MSCs cultured in TC plate, AggreWell, and suspension. At days 3–7, cells formed floating clusters in suspension, whereas the control cells were detached and released from generated clusters

Differentiation into the DE lineage was performed on WJ-MSCs (P2–P4) in triplicate, as described by Pagliuca et al. [18], with major modifications to suit the developmental stage of WJ-MSCs. For RNA extractions and the time-point differentiation profile, cells were harvested as described in the prospective study (Fig. 1a) until the end of each experiment. On the first day of differentiation, subcultured WJ-MSCs (70 % confluent) were dissociated into single cells and resuspended in Differentiation Media A. For the generation of spheroid structures, cells (1.8 × 10^6^) were added to a well of the eight-well AggreWell Plate (Stem Cell Technologies) and incubated at 37 °C in a 5 % CO_2_ incubator [19, 20]. Each well contained 1200 microwells, and accordingly each individual cell cluster was generated from 1500 cells. After 24 hours, the spheroids were harvested, washed with 1× PBS, and resuspended in fresh Differentiation Media A. The cells were then transferred into ultra-low adherence six-well plates (Corning) at a lower density, about 300–400 cells per well, in order to avoid spheroid fusion. On day 3, the medium was changed to Differentiation Media B and the cell clusters were incubated for an extra 4 days with media change every 2 days (Fig. 1a).

The constitution of the media used in the directed differentiation was similar to that used by Vegas et al. [21] with major modifications. Differentiation Media A: MCDB131 media was supplemented with 8 mM d-(+)-glucose, 14.6 mM NaHCO_3_, 1 % fatty acid-free BSA, 2 mM Glutamax, 1 % Pen/Strep (Invitrogen), 1:200 ITS-X in PBS, 250 μM ascorbic acid, 50 ng/ml KGF/FGF-7 (R&D Systems), 2 μM retinoic acid, 0.5 μM cyclopamine-KAAD (Calbiochem), 2 μM LDN193189 hydrochloride (Sigma), and 0.25 μM phorbol 12,13-dibutyrate (Sigma). Differentiation Media B: MCDB131 media was supplemented with 20 mM d-(+)-glucose, 20.9 mM NaHCO_3_, 1 % fatty acid-free BSA, 2 mM Glutamax, 1 % Pen/Strep, 0.5× N_2_ supplement, 0.5× B27 supplement without RA, 1:200 ITS-X in PBS, 250 μM ascorbic acid, 2.5 μg/ml heparin, 0.5 μM DAPT, 0.5 μM Alk5ii, 1 μM triiodiothyronine NA salt, 50 ng/ml EGF (R&D Systems), 1 μM LDN193189, 5 mM nicotine amide, 50 ng/ml Exendin-4 (R&D Systems), and 50 nM phorbol 12,13-dibutyrate.

#### Immunofluorescence assay

Immunofluorescence assays were performed on sectioned cell clusters differentiated into DE at days 3 and 7 using antibodies directed against the ME marker BraT and the DE markers CXCR4, FoxA2, and Sox17. The cryosection processing procedure was adopted from Gomes et al. [22] with minor modifications. The harvested cell clusters were washed with 1× PBS and fixed in 10 % formalin solution for 30 min at room temperature. The EBs were then rehydrated in 1× PBS for 15 min, followed by 30-min sequential incubation in a serial of sucrose solutions (10, 20, and 30 % in PBS). The sucrose solution was removed and the EBs were placed on the mold. OCT was slowly added to the mold to facilitate the assembling of cell aggregates at the center of the mold. The mounted cell cluster–OCT blocks were frozen at –80 °C for further cryosectioning using Bright OTF 5000 Cyrostat (Hacker Instruments). The sectioned spheroids were mounted onto glass slides, heat dried for 10 min to remove the OCT, and then directly used for immunofluorescence assay. For immunostaining, the slides were washed extensively with PBS and then incubated overnight with the conjugated antibodies. The slides were washed 24 hours later with PBS and mounting buffer containing Hoechst for nuclei stain. Preconjugated anti-CXCR4 (CD184) antibody was purchased from BD Pharmingen and used to detect the DE surface marker protein. Anti-human-Sox17 and FoxA2 (BD Pharmingen) were conjugated with Alexa Fluor 592 using APEX Antibody Labeling Kits (Invitrogen) as described by the manufacturers. Fluorescent and phase-contrast images were captured using Confocal Laser-Scanning microscope (LSM 710; Zeiss) as described previously [23]. Additional file 1: Table S1 presents the list of antibodies used in the study.

#### RNA extraction, cDNA synthesis, and qRT-PCR reactions

Total RNA was extracted from the cells using the Total RNA purification Kit (Norgen Biotek, Canada) in accordance with the manufacturer’s protocol. First-strand cDNA was synthesized from 50–100 ng RNA by reverse transcription using the QuantiTect Reverse Transcription Kit (Qiagen Inc., USA). Quantitative real-time PCR (qRT-PCR) reactions were performed as described previously [24, 25]. Primer pairs with equivalent efficiencies (Additional file 1: Table S2) were selected from Primer Bank [26] or were designed using primer3web (http://primer3.ut.ee/) [27] and primer-BLAST tools (http://www.ncbi.nlm.nih.gov/tools/primer-blast/) [28]. qRT-PCR was performed on the ABI7900 system (Applied Biosystems, USA) using SDS software. Relative gene expression was calculated using the comparative Ct method as described previously [29, 30]. Results were normalized to the Geo-mean of GAPDH, beta actin, and 18S Ct values, and averages ± SEM are shown expressed relative to control or day 0 undifferentiated cells, as indicated. In order to examine the DE specification, focused PCR-based array plates (PAHS-081y; Qiagen, Biosciences) were utilized. Using SYBR Green-based qRT-PCR technology in accordance with the manufacturer’s protocol, quantitative analysis of gene expression from differentiated day 7 WJ-MSCs were compared with their counterpart undifferentiated cells at day 0.

#### Flow cytometry analysis

Flow cytometric analyses were performed as described previously [1]. A small fraction of undifferentiated, day 0, and DE-derived spheroids, day 7, were washed with 1× PBS, and then enzymatically dissociated in 1 μg/ml collagenase type A in a 37 °C incubator. The enzyme was inactivated with culture media containing 10 % FBS, and cells were washed with 1× PBS and suspended in FACS buffer. Single cell suspensions were passed through 50–70 mm cell strainers. Single cells were incubated with fluorescent-conjugated CXCR4-PE antibody, diluted in FACS buffer (1:100) for 45 min, and then were washed three times in FACS buffer. Flow cytometry records were assembled as described previously [1] using a FACS Canto Flow Cytometer device and FACS Diva (BD Biosciences) software. The excitation and emission spectra were 530 nm and 590 nm, respectively. The cells were scattered at SSC vs CxCR4-PE and SSC vs FSC. The background was estimated using unstained cells with PBS or mouse IgG comparable with the isoform used with the experimental CXCR4-PE antibody.

#### Statistical analyses

In this study, WJ-MSC differentiation experiments with at least three independent cultures were performed. Statistical significance was estimated with a one-tailed Student’s *t* test assuming equal variance and the error is SEM (*P <* 0.05) [25].

## Results

### WJ-MSCs acquire defined spherical structures in 3D culture conditions

Studies have shown that the generation of embryonic bodies from human embryonic stem cells (hESCs) and human induced pluripotent stem cells (hiPSCs) is a convenient inductive step leading to downstream differentiation into the three germ layers depending on the cultural conditions [31–33]. We tested this concept on WJ-MSCs. As we have reported previously, WJ-MSCs were grown as a flat monolayer and exhibited an elongated spindle-shape fibroblast-like morphology when cultured on polystyrene tissue culture plates (Fig. 1b) [1]. Interestingly, the WJ-MSCs spontaneously generated homogeneous cell clusters upon culturing in the differentiation media using the micro-AggreWells and subsequent cultivation in low attachment plates (Fig. 1b). We also observed that the generated cell clusters are highly reliant on the initial seeded cell numbers. Our data showed that clustering of 1500 cells is sufficient to obtain spheroids with high quality and differentiation outcome (Fig. 1b, days 0 and 1; data not shown). After 24 hours, transferring the cell clusters to low-adherence plates representing suspension cultural conditions (Fig. 1b, days 3 and 7) gave them an appearance suggesting the development of normal systematized aggregate structures. In contrast, the clustered cells, using regular growth medium containing FCS, leaned toward attachment to the bottom of the low-adherence plates, where diffusions of individual cells from the clustered EBs were observed (Fig. 1a, control day 3).

### Sequential generation of ME and DE lineages

WJ-MSCs have developmental plasticity shown by their ability for multilineage differentiation [2, 34, 35]. The observed low differentiation efficiency of WJ-MSC monolayer cultures is most likely due to improper generation of DE, which is an essential step toward the potential generation of specialized organs such as the digestive track, liver, and pancreas [12, 36–39]. In order to overcome these limitations, we tested the hypothesis that mimicking embryonic developmental stages through the generation of 3D cell culture in a chemically defined serum-free media may enhance efficient DE differentiation.

**Fig. 2:**
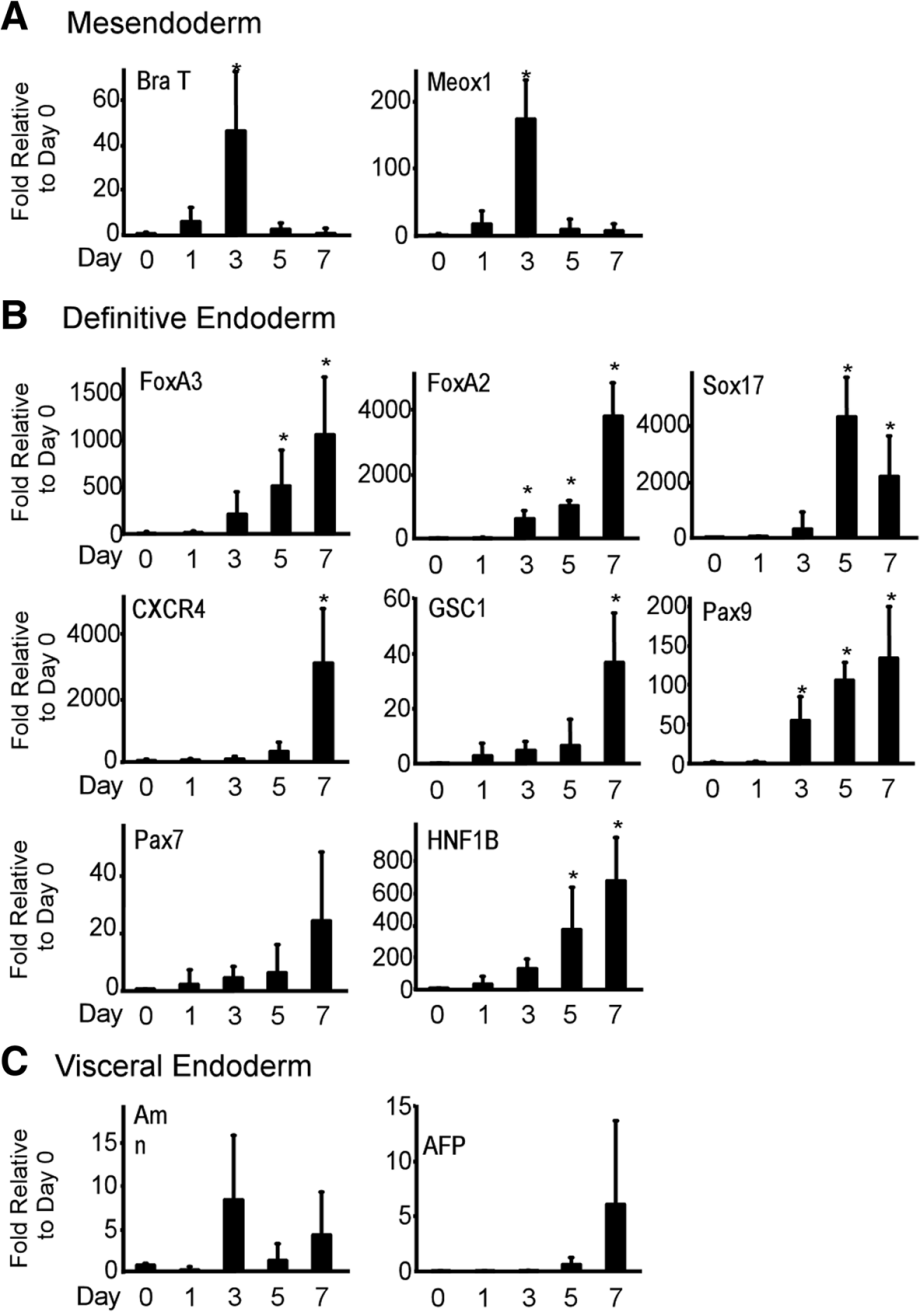
ME and DE lineage specifications. qRT-PCR was performed for the indicated genes on a time course during WJ-MSC differentiation into (**a**) ME, (**b**) DE, and (**c**) visceral endoderm. Differentiation was performed using the described experimental protocol described in Methods. Results were normalized to the Geo-mean of the housekeeping genes and expression relative to day 0. Data shown as mean ± SEM (*n* = 4). *P < 0.05

In the present study, cell aggregates were generated from WJ-MSCs and their commitment capacity toward the DE lineage was monitored (Methods). Under the described differentiation conditions, qRT-PCR analyses demonstrated an induction of Brachyury T (BraT) mRNA starting from day 1 and peaking at day 3, with a 40-fold increase relative to undifferentiated cells. Similarly, the expression levels of mesenchyme homeobox 1 (Meox1) were gradually increased and peaked at day 3 of differentiation, suggesting commitments toward primitive streak and early ME lineages (Fig. 2a) [40]. Notably, the gene expression profiling revealed that the DE specification is initiated at day 3 concomitant with a decline in the mesendodermal markers. The transcript levels of the early DE genes, Forkhead Box A3 and A2 (FoxA3 and FoxA2), emerged at day 3 and peaked at day 7 (Fig. 2b); whereas expression of the Sex Determining Region Y-box 17 (Sox17) gene peaked at day 5, with a 4000-fold increase in mRNA expression levels relative to undifferentiated cells. The chemokine (C-X-C motif) receptor 4 (CXCR4) and Goosecoid homeobox (GSC1) mRNAs were significantly enriched at day 7 (Fig. 2b). Interestingly, the increase in expression levels of the bona-fide DE markers ranged between 50-fold and 4000-fold relative to undifferentiated cells, suggesting that our approach using 3D and chemically defined differentiation conditions has successfully enriched the DE lineage from WJ-MSCs.

Next, we examined the developmental progression of the differentiated cells toward the formation of gut tube; accordingly, the transcript levels of the early posterior foregut markers Paired Box 9 (Pax9), Hepatic nuclear factor 1 β (HNF1β), and Paired Box 7 (Pax7) were studied. The transcripts of these genes were upregulated at day 3 and peaked at day 7, simultaneously with the DE markers. However, their expression levels were low, ranging between 10 and 15 %, compared with that observed for the DE genes, likely due to a possible commitment of a small cell fraction toward early posterior foregut lineage and/or the existence of these genes at lower profile in DE cells (Fig. 2b) [40, 41].

Because WJ-MSCs originate from umbilical cord tissue and in order to exclude possible generation of extra-embryonic visceral endoderm lineage, we assessed the gene expression of this particular developmental stage. qRT-PCR analysis exhibited a nonsignificant regulation in the transcripts of the visceral endodermal genes, amnion associated transmembrane protein (Amn) and alpha-fetoprotein (AFP), during the time course of differentiation (Fig. 2c). The chronological gene expression dynamics thus particularly recapitulate those in ME and DE lineage development.

**Fig. 3:**
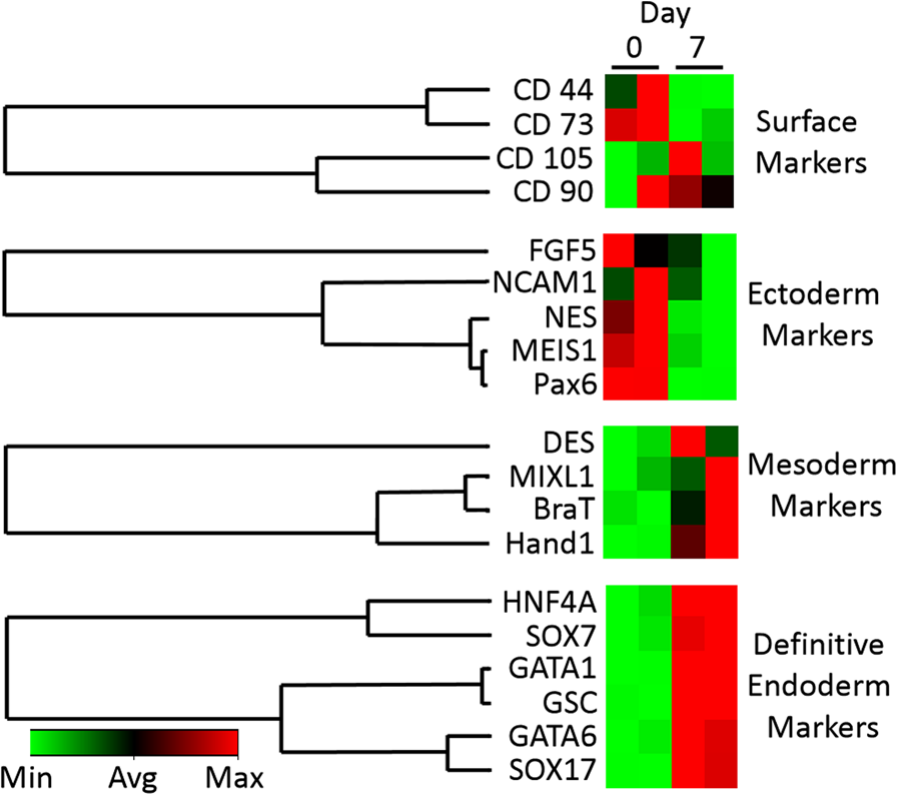
Differential gene expression of undifferentiated and differentiated WJ-MSCs. Heatmap gene expression regulations at days 0 and 7. Alteration in WJ-MSC surface markers, loss of ectodermal markers, and upregulation of the DE markers suggest a directed differentiation toward the later lineage

Targeted microarray analysis was generated to distinguish the gene expression signature of undifferentiated versus day 7 differentiated WJ-MSCs (Fig. 3). In accordance with our previous study, downregulation of the WJ-MSC surface markers CD44 and CD73 was observed at day 7 of differentiation, supporting our previous conclusion that these two proteins are reliable stemness markers for WJ-MSCs [1]. On the contrary, CD105 and CD90 were upregulated in response to DE generation (Fig. 3). Additionally, genes known to be important regulators of the ectodermal differentiation such as Pax6, MEIS1, and NCAM1 were depleted, suggesting that the cellular specification is waived from the ectoderm formation using the described protocol (Fig. 3). Remarkably, as described previously in Fig. 2, a nonconsistent regulation of the expression of the mesodermal genes MixL1, BraT, and Hand1 was observed, marking the initiation of DE lineage specification. This notation was further confirmed with the increase in the expression of the DE genes such as SOX17, GSC1, and GATAs, which are landmark genes for the DE lineage (Fig. 3).

### Efficient generation of DE cells from WJ-MSCs

Gene expression analysis revealed a distinct DE signature at day 7 of WJ-MSC differentiation. In order to corroborate the qRT-PCR analysis and evaluate the differentiation efficiency, we ascertained the protein expression of the DE markers by immunolocalization and flow cytometric analysis.

**Fig. 4:**
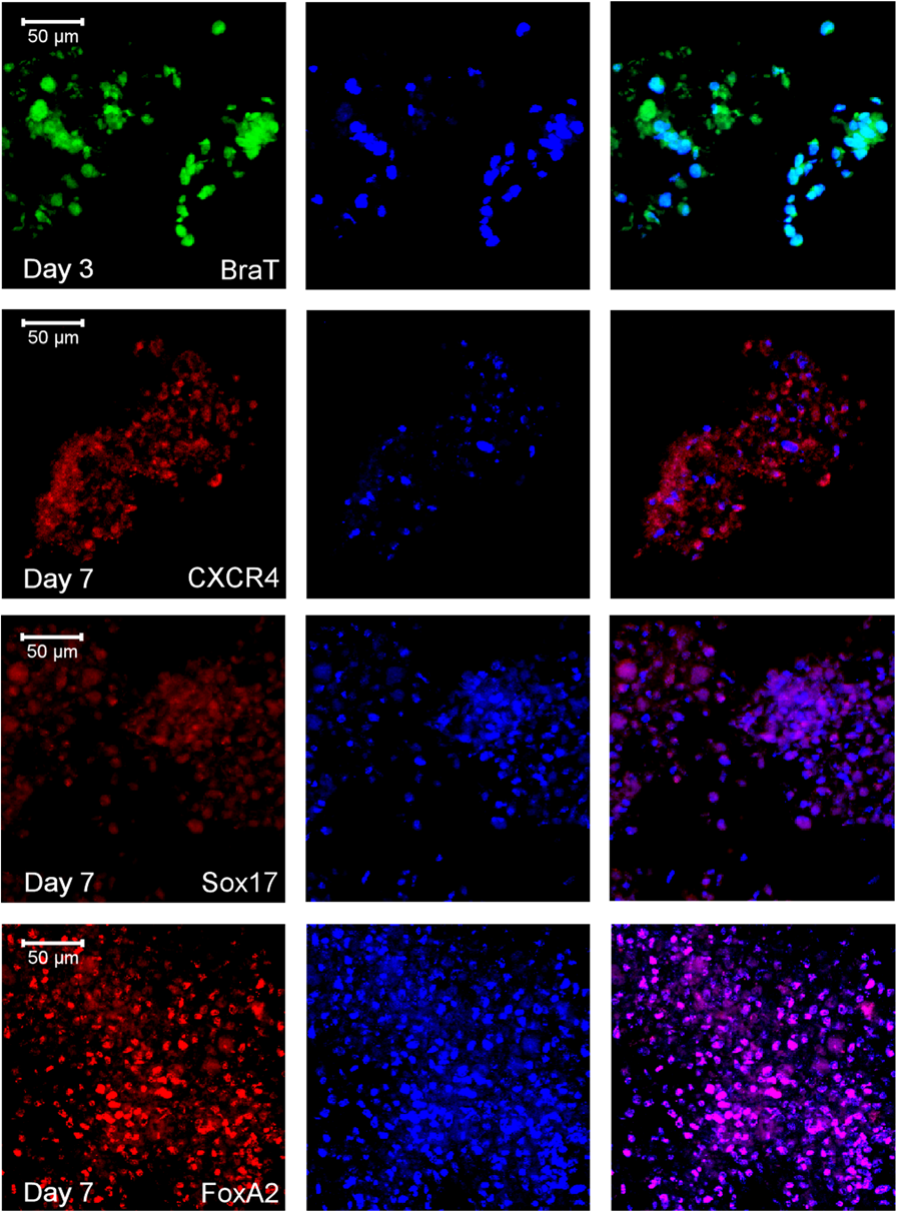
Immunofluorescence of differentiated WJ-MSCs. Confocal laser representative images for differentiated WJ-MSCs at days 3 and 7 as indicated. Immunofluorescence assays using the APEX-labeling system for conjugating primary antibodies (BraT-Alexa 488, Sox17-Alexa 594, and FoxA2-Alexa 594). CXCR4 antibodies are PE congregated. Magnification × 400

Immunofluorescence with anti-BraT antibodies detected its nuclear protein localization at day 3 of differentiated WJ-MSCs (Fig. 4, Additional file 1: Figure S1), which was dramatically lost at day 7 (data not shown). CXCR4 protein was detected predominantly at the cell membrane of differentiated WJ-MSCs, whereas Sox17 and FoxA2 proteins were expressed at the nuclei of the generated DE cells at day 7. The chronological elevation in the expression of FoxA2 proteins at day 7 is consistent with the reduction of the ME marker BraT, and the increase of the DE markers CXCR4 and Sox17 is indicative of differentiation toward the later lineage (Fig. 4, Additional file 1: Figure S1).

**Fig. 5:**
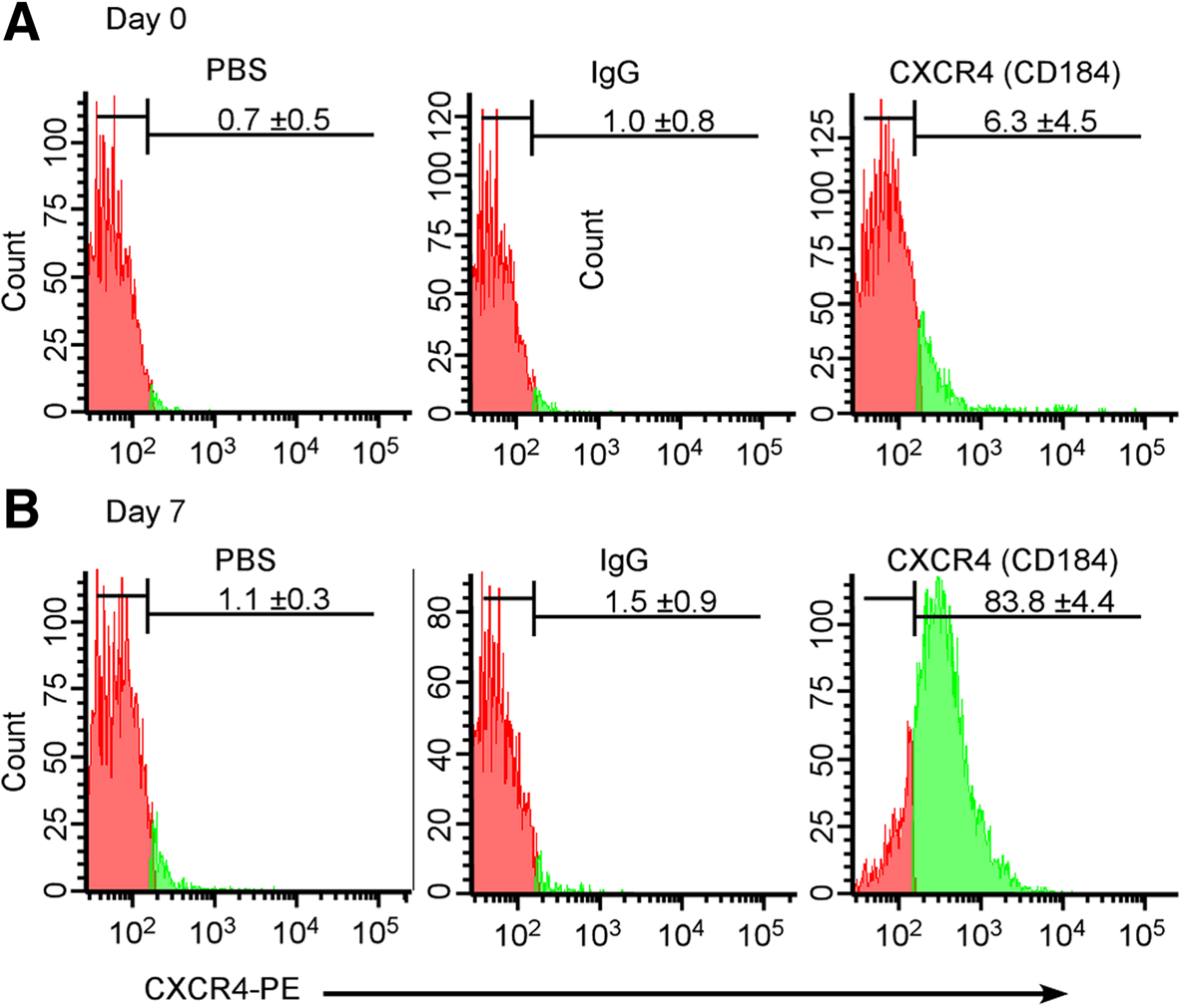
Flow cytometric analysis of the DE bona-fide marker CXCR4 at **a** day 0 and **b** day 7. Unlike the differentiated WJ-MSCs, the undifferentiated cells were negative to the expression of the surface protein CXCR4. Examples of flow cytometric images from a representative experiment. Data shown as mean ± SEM (*n* = 3)

Immunoreactivity analysis revealed a high expression of the DE surface protein CXCR4, which inspired us to investigate the differentiation efficiency using flow cytometry. The differentiation protocol resulted in an enrichment of DE; where 83 ± 4.4 % of the total cell count were positive to CXCR4 protein at day 7, relative to cells treated with comparable IgG or PBS, which showed minor backgrounds for CXCR4 expression (1.1–1.5 %, Fig. 5). In accordance with previous studies performed on adult MSCs, undifferentiated WJ-MSCs cultured on a monolayer showed a statistically nonsignificant minor expression of CXCR4 (6.3 ± 4.5 % Fig. 5) [42, 43]. Adult fibroblast cells were also subjected to the same differentiation conditions. At day 7, these cells showed nonspecific expression for CXCR4 relative to IgG-treated and PBS-treated cells: 17.6 ± 1.8 and 9.4 ± 7.2, respectively (Additional file 1: Figure S2).

**Fig. 6:**
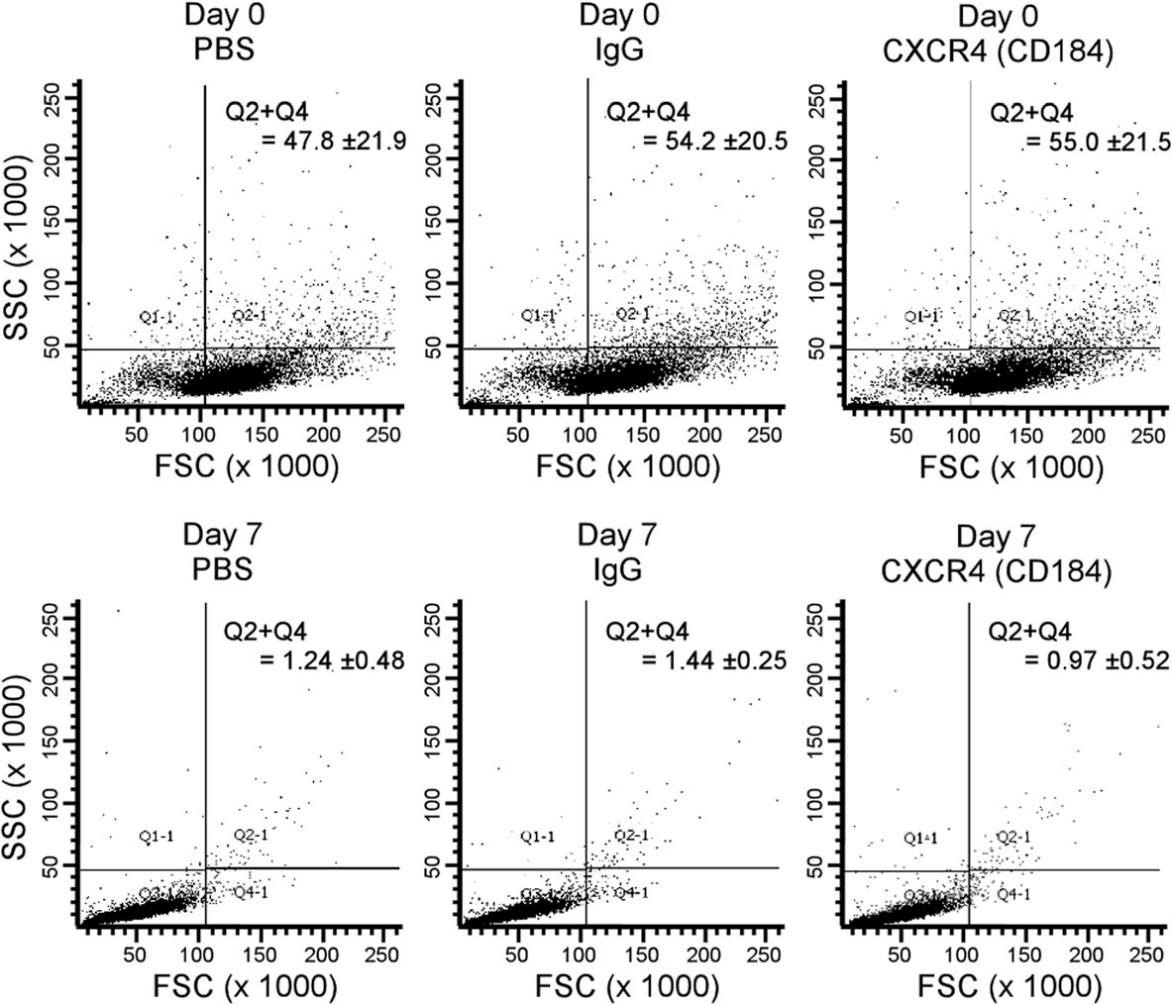
Flow cytometric side-scattered (*SSC*) vs forward-scattered (*FSC*) pattern of undifferentiated and generated DE. At day 0, a heterogeneous population of WJ-MSCs was observed, whereas the differentiated cells showed a homogeneous population characterized by small-sized cells with less granulation. Examples of flow cytometric images from a representative experiment. Data shown as mean ± SEM (*n* = 3)

Scatter-dot plots analysis from undifferentiated, day 0, and differentiated, day 7, WJ-MSCs were generated. Undifferentiated WJ-MSCs exhibited high forward-scatter (FSC) and moderate to high side-scatter patterns (Fig. 6, day 0), whereas at differentiation day 7 approximately 99 % of the generated DE cells demonstrate low scatter parameters (Fig. 6), implying a small size and less granularity. These data support previous reports showing that WJ-MSCs consist of a heterogeneous population [44, 45], and reflect efficient differentiation toward a homogeneous population of DE cells, which are characterized with small-sized cells and less granularity [46]. Taking these observations together, the implemented 3D differentiation protocol using chemically defined media efficiently induced the generation of DE cells.

## Discussion

In order to develop a protocol for stem cell differentiation toward clinically relevant cell types, the current strategy is mimicking the cell signaling events associated with the embryonic developmental process for the lineage of interest [8, 47, 48]. Nevertheless, in-vitro cell differentiation does not reflect a stepwise developmental progression but gives a broad range of outcome lineages [49]. Accordingly, the development of a protocol that significantly improves a targeted cell type is of particular interest. Directed differentiation methods utilize chemically well-defined small molecules, which regulate cell-signaling pathways, resulting in cellular differentiation toward a particular lineage [25, 50, 51]. In addition, studies have shown that the generation of 3D spheroid structures enhances the cellular programming potential. These approaches have been successfully applied to hESCs and hiPSCs [51–53], but have yet to be applied to other pluripotent cell types, including WJ-MSCs.

In this study, we implemented an optimized two-stage protocol directed to a stepwise differentiation of WJ-MSCs toward the DE cell population, the progenitor for the development of gut tube, pancreatic, and hepatic cells [8, 38, 54]. For efficient differentiation, WJ-MSCs were aggregated in suspension to generate uniform clusters of differentiated cells mimicking the formation of embryonic bodies observed with ESCs or iPSCs. Initially, we applied the recently described protocol used to differentiate hESCs and hiPSCs by Pagliuca's group [18]. Under 3D conditions, WJ-MSCs were cultured for 3 days in a differentiation media supplemented with Activin and WNT3a activators, followed by 3-day incubation in a media containing retinoic acid (RA) and keratinocyte growth factor (KGF), in the presence of sonic hedgehog (SHH) and bone morphogenetic protein (BMP) inhibitors. Time-course RT-PCR analysis revealed an increase in BraT and Meox1 transcripts, the primitive streak/mesendodermal (PS/ME) markers [55], following the second treatment, suggesting that Activin/Wnt signaling failed to induce PS/ME lineages in WJ-MSCs (data not shown). Accordingly, we eliminated the Activin/Wnt activator treatment step in the subsequent experiments (Fig. 1). Activin A has been defined to trigger PS/ME lineages in both ESCs and iPSCs [41, 56–58]; however, it maintains multipotency in MSCs [59]. The differences in the role of Activin A are related to the differences in stem cell origins and their developmental stages. Further, undifferentiated WJ-MSCs were reported to secrete Activin A as an immunosuppression agent reducing natural killer-mediated IFN-γ production [60, 61], which supports our results that Activin A signaling is not required for WJ-MSC differentiation.

Interestingly, our results revealed a combination of the RA/KGF inducers and SHH/BMP signaling inhibitors to be sufficient to enhance WJ-MSC-mediated PS/ME lineage inductions. Reports have indicated that each signaling molecule is involved in a particular lineage generation; for example, KGF induces MSC differentiation into sweat gland-like cells [62]. RA and SHH molecules were reported to mediate osteogenesis and abolish adipogenesis in MSCs independently [63–65]. However, synergistic RA and SHH signaling promotes the generation of sensory neurons [66]. On the contrary, BMP signaling acts as an inhibitor for the early stages of human ESC-mediated DE development [67, 68]. Consequently, cells respond differentially to multiple changes in the extracellular environment [69, 70]. It is important to note that the differentiation protocol used in this study is serum free. Thus, no extracellular signaling molecules were involved in the generated PS/ME lineages other than the interplay between the four described signaling pathways.

WJ-MSC-mediated DE formation was enhanced by thyroid hormone, exendin-4, and EGF; inhibition of both TGF-β and Notch pathways was also effective to enhance the yield of this lineage. Cvoro et al. reported that during DE induction from hESCs and hiPSCs, thyroid hormone suppresses notch-signaling genes through the activation of the Kruppel-like factor KLF-9 [71, 72]. Both exendin-4 and EGF direct the DE differentiation toward primitive gut tube endoderm, and enhance proinsulin biosynthesis and expansion of pancreatic progenitor at later stages of hESC and hiPSC differentiation [18]. Recently, Nekoei et al. [73] reported the generation of insulin-producing cells from WJ-MSC cultured in a serum-dependent differentiation containing exendin-4 [24], yet the efficiency of insulin induction was relatively low most likely due to absence of the stepwise differentiation process. Similarly, Kadam et al. [74] reported a low yield of pancreatic β-cell-like clusters generated from monolayer cultures of placenta-derived MSCs. Inhibition of TGF-β receptor ALK5 was reported to mediate human ESC differentiation into the early gut and induce Pdx1-expressing endodermal cells [69, 75].

Unlike the traditional 2D adherent cell cultures, 3D cellular clustering provides the cells with physiological conditions similar to those occurring in embryos. Remarkably, WJ-MSCs generated stable spheroidal bodies upon culturing in AggreWell plates, which showed an improved differentiation capacity into DE lineage. In both ESCs and iPSCs this technique has been proven to efficiently mediate the differentiation toward different developmental lineages [8, 24, 25], but it has not been tested in WJ-MSCs. Notably, differentiating WJ-MSCs in monolayer cultures toward insulin-producing cells resulted in the generation of cell aggregates; while remaining attached to the plate surface, cells within the cluster-like morphology showed a significant differentiation property [73, 76].

## Conclusions

We established a differentiation method that induces DE cells from WJ-MSCs with high efficiency. The 3D cultural environment and serum-free media administered with controlled extracellular signaling pathways resulted in more than 85 % enrichment of DE cells, the progenitor germ layer for pancreatic and hepatic cells. Accordingly, in addition to their advantage over other stem cells, WJ-MSCs provide an excellent platform for DE and downstream lineage differentiation.

## Additional file

Additional file 1: Table S1. Presenting primary antibodies used for flow cytometry characterization of differentiated cells, Table S2 presenting oligonucleotide sequences of primers utilized for real-time qRT-PCR, Figure S1 showing immunofluorescence of the differentiated WJ-MSCs as confocal laser representative images for differentiated WJ-MSCs at days 3 and 7 as indicated, and Figure S2 showing flow cytometric analysis of the definitive endoderm bona-fide marker CXCR4 at day 7. (PDF 519 kb)

## Abbreviations

3D: Three-dimensional
AFP: Alpha-fetoprotein
Amn: Amnion-associated transmembrane protein
BraT: Brachyury T
BSA: Bovine serum albumin
CXC4: Chemokine (C-X-C motif) receptor 4
DE: Definitive endoderm
DMEM: Dulbecco’s modified Eagle medium
EGF: Epidermal growth factor
ESC: Embryonic stem cell
FACS: Fluorescence-activated cell sorting
FoxA2: Forkhead Box A2
FoxA3: Forkhead Box A3
GSC1: Goosecoid homeobox
HNF1β: Hepatic nuclear factor 1 β
iPSC: Induced pluripotent stem cell
ITS-X: Insulin–transferrin–selenium–ethanolamine
KGF/FGF-7: Keratinocyte growth factor, fibroblast growth factor-7
KLF-9: Kruppel-like factor
ME: Mesendoderm
Meox1: Mesenchyme homeobox 1
NaHCO_3_: Sodium bicarbonate
Pax7: Paired Box 7
Pax9: Paired Box 9
PBS: Phosphate-buffered saline
PSC: Pluripotent stem cell
qRT-PCR: Quantitative real-time polymerase chain reaction
Sox17: Sex Determining Region Y-box 17
WJ-MSC: Wharton’s Jelly-derived mesenchymal stem cell
