# Supplementary material for: Defined three-dimensional culture conditions mediate efficient induction of definitive endoderm lineage from human umbilical cord Wharton’s jelly mesenchymal stem cells
Source: Stem Cell Res Ther. 2016 Nov 16;7:165. doi: 10.1186/s13287-016-0426-9 (PMC5111269; doi:10.1186/s13287-016-0426-9)

Supplementary Information

Defined three-dimensional culture conditions mediate efficient induction of Definitive Endoderm lineage from human umbilical cord- Wharton's Jelly- Mesenchymal Stem Cells.

Ashraf Al Madhoun [1,*, #], Hamad Ali [1,2,*], Sarah AlKandari [1], Valerie Lopez Atizado [1], Nadeem Akhter [1], Fahd Al-Mulla [3] and Maher Atari [4].

Table 1: Primary Antibodies used for Flow Cytometry characterization of differentiated cells

Antibody	Conjugate	Manufacturer	Catalogue Number
CXCR4	PHYCOERYTHRIN	BD Pharmingen	551966
FOXA2	--	BD Pharmingen	561580
SOX17	--	BD Pharmingen	561590
BraT	--	R&D	AF2085

Table 2: Oligonucleotide sequences of primers utilized for real-time qRT-PCR.

Genes	Forward Primer (5'-3')	Reverse Primer (5'-3')
GAPDH	GGAGCGAGATCCCTCCAAAAT	GGCTGTTGTCATACTTCTCATGG
S18	ATCACCATTATGCAGAATCCACG	GACCTGGCTGTATTTTCCATCC
Meox1	GCAGGGGGTTCCAAGGAAAT	GTCAGGTAGTTATGATGGGCAAA
Bra T	CTGGGTACTCCCAATGGGG	GGTTGGAGAATTGTTCCGATGA
AMN	GTCCCCAACACGGACTTCG	CATGTCTGAGACGGCGTGAC
AFP	AGTGAGGACAAACTATTGGCCT	ACACCAGGGTTTACTGGAGTC
FOXA2	GGAGCAGCTACTATGCAGAGC	CGTGTTCATGCCGTTCATCC
FOXA3	AGTGGAGCTACTACCCGGAG	GCTTAGAGGATTCAGGGTCATG
SOX17	TTTCATGGTGTGGGCTAAGGA	TTGTAGTTGGGGTGGTCCTG
GSC1	AACGCGGAGAAGTGGAACAAG	CTGTCCGAGTCCAAATCGC
CXCR4	ACTACACCGAGGAAATGGGCT	CCCACAATGCCAGTTAAGAAGA
FOX7	GCCAAGGACGAGAGGAAAC	GTTGGGGTAGTCCTGCATGT
HNF1b	GTGGACCGGATGCTCAGTG	GGGTCTTCATAGGGGTGCC

FIGURE LEGENDS

Supplemental Figure 1. Immunofluorescence of the differentiated WJ-MSCs. Confocal laser representative Images for differentiated WJ-MSCs at days 3 and 7 as indicated. Immunofluorescence using APEX-labeling system for conjugating primary antibodies (BraT-Alexa 488; Sox17- and FoxA2- Alexa 594), CXCR4-PE congregated. The magnification is 200X.

Supplemental Figure 2. Flow cytometric analysis of the definitive endoderm bona fide marker CXCR4 at day 7. Unlike the differentiated WJ-MSCs, human adult fibroblast subjected to the same differentiation protocol failed to express CXCR4 suggesting that specification of the experimental protocol to WJ-MSCs. Nonspecific binding to the fibroblast cell surface was observed for both IgG and CXCR4 antibodies. Examples of flow cytometric images from a representative experiment. Data are shown as mean ± s.e.m. (n = 3).

Supplement Figure 1

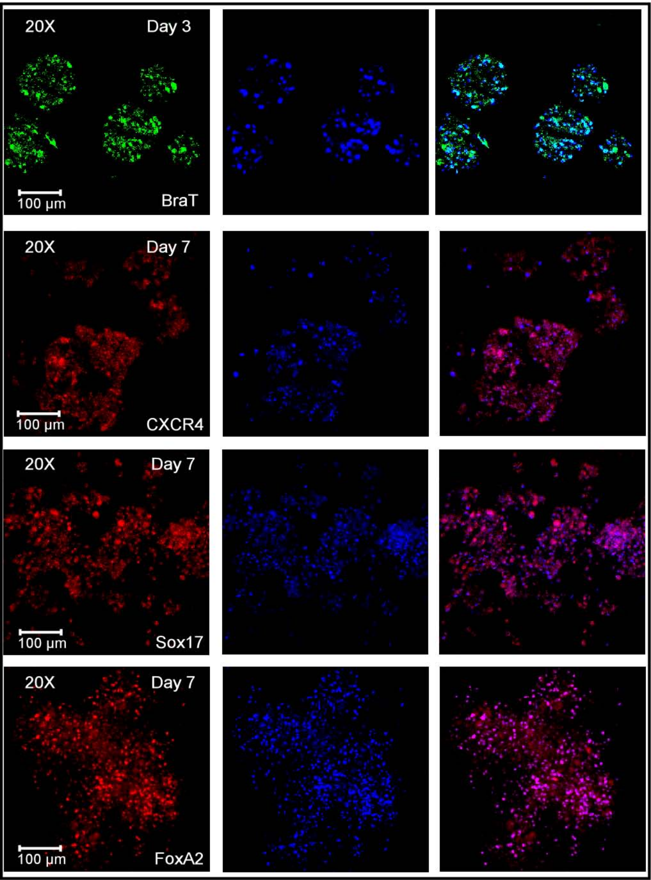

Supplemental Figure 2

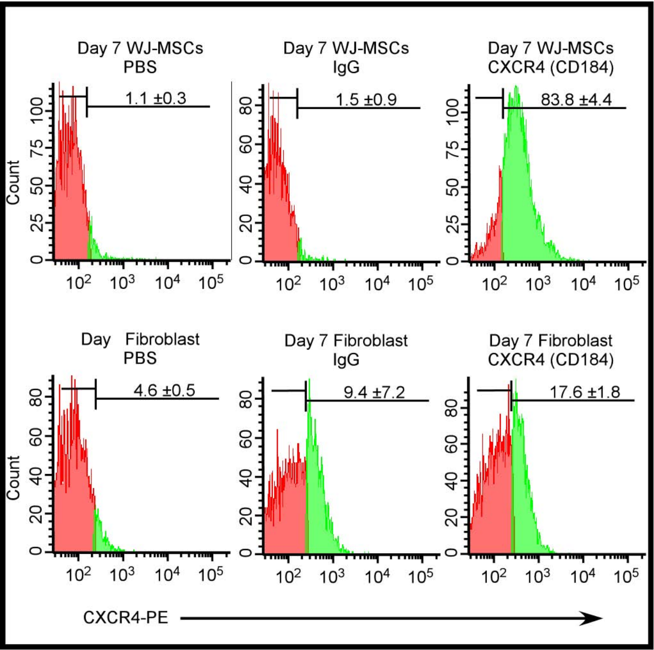

Supplement: Additional file 1: Table S1. — Presenting primary antibodies used for flow cytometry characterization of differentiated cells, Table S2 presenting oligonucleotide sequences of primers utilized for real-time qRT-PCR, Figure S1 showing immunofluorescence of the differentiated WJ-MSCs as confocal laser representative images for differentiated WJ-MSCs at days 3 and 7 as indicated, and Figure S2 showing flow cytometric analysis of the definitive endoderm bona-fide marker CXCR4 at day 7. (PDF 519 kb) [file 13287_2016_426_MOESM1_ESM.pdf]
